# Supplementary material for: Hybrid Models and Biological Model Reduction with PyDSTool
Source: PLoS Comput Biol. 2012 Aug 9;8(8):e1002628. doi: 10.1371/journal.pcbi.1002628 (PMC3415397; doi:10.1371/journal.pcbi.1002628)
Supplement: Text S4 — Complete source code for the PyDSTool package (version 0.88.120504). Includes API documentation and help files linking to web pages. This file is identical to the current public release on Sourceforge.net. (ZIP) [file pcbi.1002628.s004.zip › PyDSTool/html/PyDSTool.Toolbox.optimizers.line_search.scaled_line_search.ScaledLineSearch-class.html]

xml version="1.0" encoding="ascii"?


PyDSTool.Toolbox.optimizers.line\_search.scaled\_line\_search.ScaledLineSearch


| Home | Trees | Indices | Help | PyDSTool |
| --- | --- | --- | --- | --- |

Package PyDSTool ::
Package Toolbox ::
Package optimizers ::
Package line\_search ::
Module scaled\_line\_search ::
Class ScaledLineSearch

[hide private]

[frames] | no frames]

# Class ScaledLineSearch

source code

```
object --+
         |
        ScaledLineSearch
```

---

A simple line search, takes a point, adds a step and returns it Scales
step according to given scales of the parameters and ignores \*magnitude\*
of gradient.

(in early development and experimental only at this point)


|  |  |
| --- | --- |
| Instance Methods | [hide private] |

|  |  |
| --- | --- |
| \_\_init\_\_(self, max\_step=1, step\_mod=3, \*\*kwargs)  Needs to have :... | source code |

|  |  |
| --- | --- |
| \_\_call\_\_(self, origin, state, \*\*kwargs)  Returns a good candidate... | source code |

**Inherited from `object`**:
`__delattr__`,
`__getattribute__`,
`__hash__`,
`__new__`,
`__reduce__`,
`__reduce_ex__`,
`__repr__`,
`__setattr__`,
`__str__`


|  |  |
| --- | --- |
| Properties | [hide private] |

**Inherited from `object`**:
`__class__`


|  |  |
| --- | --- |
| Method Details | [hide private] |

|  |  |
| --- | --- |
| \_\_init\_\_(self, max\_step=1, step\_mod=3, \*\*kwargs)  *(Constructor)* | source code |

```
Needs to have :
- nothing
Can have :
- max_step: a maximum step control, a scalar or vector to restrict step size
in each direction (default 1)
- step_mod: a factor to divide the step when back-tracking (default 3)
- max_reduce_fac: max_step divided by this is the smallest step that will be tried (default 2000),
```

Overrides: object.\_\_init\_\_

|  |  |
| --- | --- |
| \_\_call\_\_(self, origin, state, \*\*kwargs)  *(Call operator)* | source code |

```
Returns a good candidate
Parameters :
- origin is the origin of the search
- state is the state of the optimizer
```

  


| Home | Trees | Indices | Help | PyDSTool |
| --- | --- | --- | --- | --- |

|  |  |
| --- | --- |
| Generated by Epydoc 3.0.1 on Fri May 4 15:24:09 2012 | http://epydoc.sourceforge.net |
